# Pindborg tumor in early childhood: a rare tumor in the youngest patient reported to date

**DOI:** 10.1080/23320885.2022.2031201

**Published:** 2022-02-02

**Authors:** Brian W. Starr, Elizabeth A. Lax, Angelo A. Leto Barone, Raquel M. Ulma, Brian S. Pan, Haithem M. Elhadi Babiker

**Affiliations:** Division of Plastic Surgery, Cincinnati Children’s Hospital Medical Center, Cincinnati, OH, USA

**Keywords:** Intraosseous CEOT, Pindborg tumor, pediatric odontogenic tumor, pediatric mandible tumor, odontogenic neoplasm

## Abstract

Pindborg tumor is a benign expansile and slow growing odontogenic tumor that occurs mainly in adulthood. Limited management data exist for its treatment in young patients. We report the case of a 5-year-old patient and provide recommendations for the care of pediatric patients diagnosed with this rare odontogenic tumor.

## Introduction

Calcifying epithelial odontogenic tumor (CEOT), also known as Pindborg tumor, was described by Danish oral pathologist Jens Pindborg in 1955 [1]. This benign odontogenic neoplasm is slow growing and expansile. Among all types of odontogenic tumors, CEOT accounts for only 1% of cases [2,3]. Primarily intraosseous, the tumor typically occurs in the posterior mandible, with over half of the described cases being associated with tooth impaction [1,4,5] and most patients being asymptomatic. An extraosseous variant, usually found in the anterior gingiva, has also been reported [6].

There are approximately 350 cases of CEOT reported in the literature. None of the 350 cases have occurred in a patient younger than 8 years of age. A recent review by Chrcanovic et al. analyzed 339 cases of CEOT. The study found that the mean age of presentation of CEOT patients is 38.1 years and identified that the highest tumor recurrence rate was associated with treatment by enucleation and curettage [7]. Although CEOT’s are frequently benign, malignant transformation has been described [8–10]. While the occurrence of CEOT is rare, its radiographic and histopathologic features are well described. The radiographic features of CEOT include a unilocular or multilocular radiolucency, with radiopaque flecks within the central radiolucent area. Its unique radiographic features are often likened to the appearance of ‘driven snow’ [1,3]. The tumor histology is characterized by the presence of polygonal epithelial tumor cells, calcifications, and eosinophilic deposits resembling amyloid [1,11]. Concentric rings of basophilic calcification, called Liesegang rings, are found within the amyloid-like deposits [12].

Given the paucity of data that exists on CEOT in the pediatric population, there is no consensus on its diagnosis, surgical treatment, tumor surveillance or timing of reconstruction. Herein, we present a case of an intraosseous CEOT in a 5-year-old child, and share our algorithmic approach for its management in the pediatric population.

## Case description

**Figure 1.**
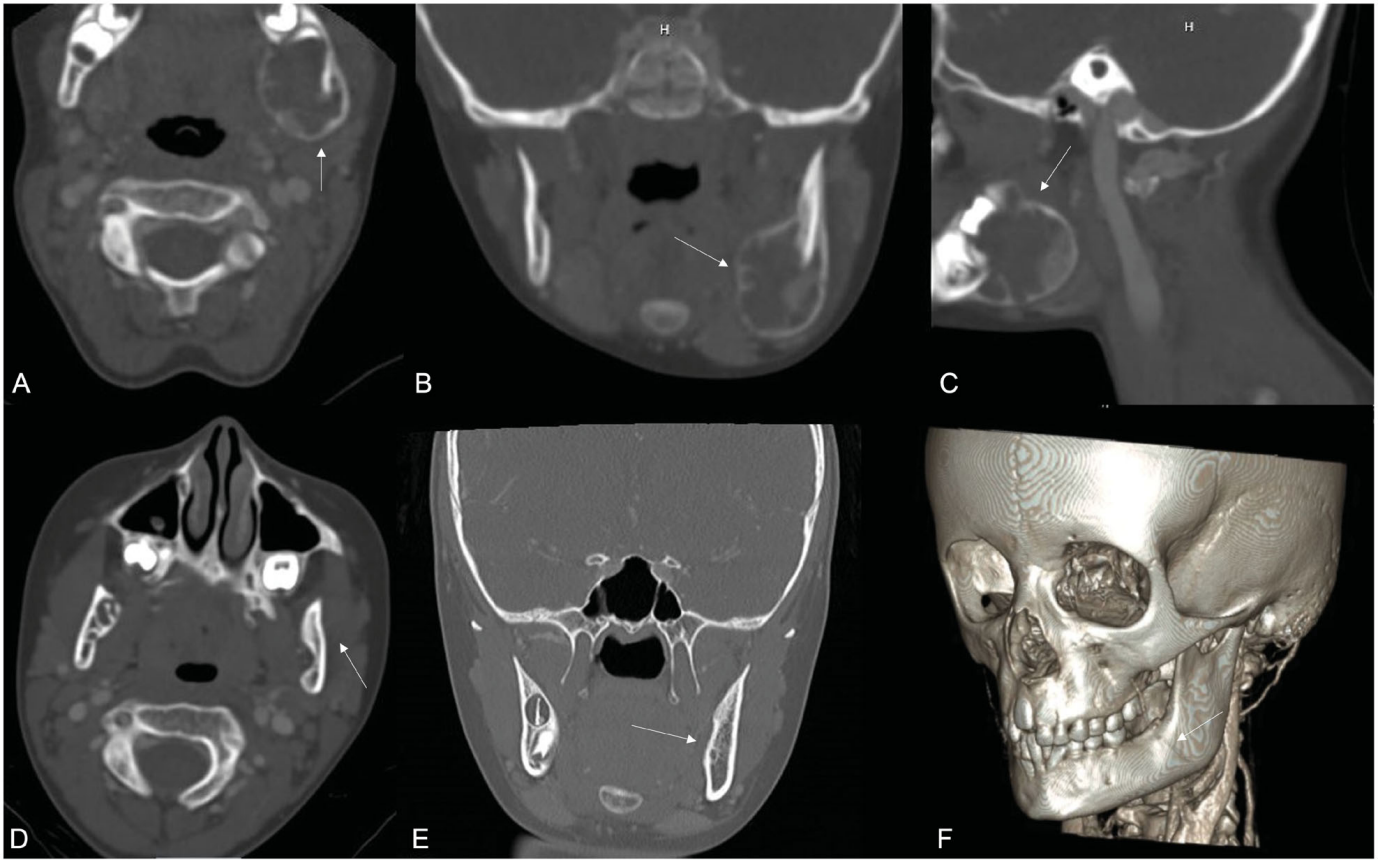
Top row: preoperative CT neck images demonstrating 2 × 2 × 3 cm expansile lesion of the left posterior mandible, mixed radiolucent-radiopaque with trabeculations. (A) axial view, (B) coronal view, (C) sagittal view. Bottom row: postoperative face CT performed three years after surgery. Image demonstrates preservation of the left mandible cortices with normal contours in axial (D), coronal (E), and lateral (3D image for better representation, F) showing no evidence of recurrence.

The patient was an otherwise healthy 5-year-old female who presented to our institution for a second opinion regarding management of a mandibular mass. She reported 2 weeks of left lower mandibular pain, followed by a week of progressive left facial swelling. Laboratory studies (CBC, sedimentation rate, CRP, etc.) were unremarkable. Due to lack of response to empiric oral antibiotic treatment, a computed tomogram (CT) of the neck with contrast was obtained. Imaging demonstrated an expansile lesion of the left mandibular angle, measuring 2 × 2 × 3 cm. The intraosseous mandibular lesion was mixed radiolucent-radiopaque, with trabeculations (Figure 1(A–C)).

Clinical exam revealed mild left-sided facial swelling without signs of infection. Intraorally, bony expansion of the left posterior mandible was noted. There was no tooth mobility and her oral mucosa was intact. Her clinical findings were suggestive of an odontogenic tumor. Given the patient’s age, clinical findings of asymptomatic expansion, posterior mandibular location, and the CT findings, a benign odontogenic pathology was suspected. Given the patient’s age and the low risk of malignancy in the presentation, we decided to proceed directly to definitive treatment. Tumor enucleation and peripheral ostectomy was planned.

The patient was taken to the operating room and the tumor was aspirated with no return of fluid. The tumor was then accessed intraorally via an incision along the left posterior mandible. Subperiosteal dissection was carried out, and a bony window was made, in an area where the tumor had thinned out the bone. The tumor was carefully enucleated and peripheral ostectomy (removal of about 1 mm of bone from all involved surfaces) was performed with a round bur. Follicles for developing teeth #18 and 19 were embedded within the tumor and were therefore removed with the specimen. The mandibular cortices and inferior border were mostly intact, and therefore no immediate bony reconstruction was performed. The defect was packed with absorbable gelatin sponge and the incision was closed in a watertight fashion.

**Figure 2.**
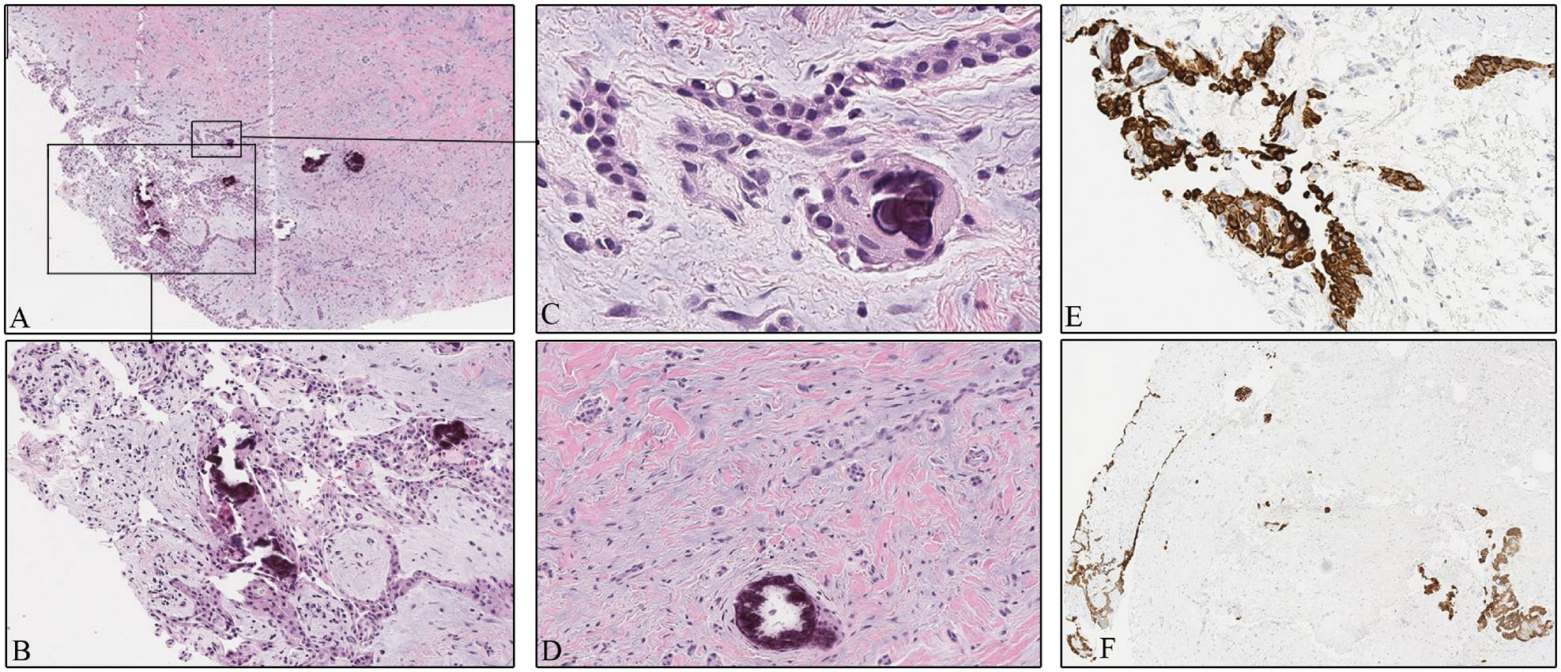
The fragmented cyst wall includes fibro-collagenous tissue (A), with scattered haphazardly arranged odontogenic islands, focally lined by an epithelial neoplastic proliferation (B, C). These are large polygonal cells with ample amounts of eosinophilic cytoplasm and prominent cell borders (G). Foci of irregular dystrophic calcifications (C, F) with concentric lamination called Liesegang rings (G), are associated with this tumor. CK19 highlights the epithelium (E, H). Small infiltrative epithelial islands are apparent focally (F, H).

Final pathology confirmed the diagnosis of CEOT. Histopathology showed large polygonal epithelial cells with ample granular and eosinophilic cytoplasm and well-defined cell borders. Nucleoli were focally present, as well as irregular calcifications and Liesegang rings (Figure 2(A–H)).

In our case, no reconstruction was performed at the time of resection. We opted to defer bone grafting to avoid obscuring the surveillance picture and to allow time for spontaneous bony regeneration. Given the low suspicion for malignancy and likely odontogenic tumor etiology in this case, we decided to pursue a semi-conservative approach. Strong consideration was given to the patient’s young age, excellent healing potential, and small bony defect size that could fill on its own. We did not bone graft any mandible defect after enucleation and peripheral ostectomy, but instead, take advantage of the pediatric healing potential. Our patient is now almost four years out from surgery, and she remains clinically and radiographically disease-free, with excellent bony regeneration and normal facial contours (Figure 1(D–F)). No adjuvant therapy, such as liquid nitrogen or other chemotherapeutic agents, was used.

## Discussion

The classic presentation for CEOT involves an asymptomatic, slow growing, expansile lesion of the posterior mandible affecting individuals in their 3rd–6th decades of life. Although rare, the peak incidence of CEOT occurs in the 5th decade [1,11,12]. Guerrisi et al. described their 15-year experience with odontogenic tumors in children and adolescents. They reported 153 pediatric odontogenic tumors, only two of which were CEOT [2]. Both patients were in their second decade of life, and they didn’t report on their treatment.

Although CEOTs are frequently benign, there are reports that describe malignant transformation [8–10]. Described surgical treatment options vary widely, from conservative enucleation and curettage, resection with negative margins, and even hemimandibulectomy or hemimaxillectomy [13,14]. Precise guidelines for the management and follow up of these patients do not exist. The CEOT recurrence rate in adults is approximately 15% [7,14–16], and a long-term follow up of 5–10 years is suggested for the adult patients. Franklin and Pindborg reported a series of 113 CEOT patients treated with enucleation and curettage with 1 year follow up and a recurrence rate of 14–30%, with an average recurrence of 14% [16]. This rate is often quoted in recent literature, and newer reports confirm the recurrence to be in a similar range (12.6–15%) [7,15]. No prospective studies have been conducted, and the recurrence rate post *en bloc* resection is unknown [8,16,17]. In a review of 339 CEOT cases reported in the literature, Chrcanovic et al. report recurrence rates of 12.6% for all CEOT lesion types, 18.8% for peripheral lesions, 11.6% for central lesions, 10.7% for clear cell variant lesions, 0% for Langerhans cell-rich variant lesions, and 42.9% in cases with known malignant transformation [7]. They also noted that the highest risk of recurrence (24.3%) was following treatment with excision and curettage. Patients undergoing either marginal resection (0% recurrence) or segmental resection (7.5% recurrence) had lower recurrence rates [7].

The main advantage of an immediate mandibular reconstruction is to provide a functional and aesthetically acceptable outcome at the time of tumor resection. A staged approach with delayed mandibular reconstruction has proven to be a viable alternative. Troulis et al. described a staged reconstructive protocol for pediatric patients undergoing treatment of mandibular tumors [18]. Their approach consists of *en-bloc* tumor resection and placement of a rigid reconstruction plate spanning the mandibular defect. Six months later, mandibular continuity is restored with secondary bone grafting. Dental prosthetic rehabilitation follows, with placement of osseointegrated dental implants at skeletal maturity.

To date, the 5-year-old patient presented herein is the youngest person reported with an intraosseous CEOT. Such a young patient presents the surgeon with several management dilemmas, including how aggressively to treat a CEOT presenting in early childhood, and how to avoid multiple operations in a young patient. Determining the need for, and timing of bony reconstruction, as well as setting up an optimal tumor surveillance plan are critical in this population. Our differential diagnosis based on clinical presentation alone initially included dentigerous cyst, odontogenic keratocyst, central giant cell lesion, osteoblastoma, and ameloblastoma. When considering the imaging findings, our differential diagnosis expanded to include other mixed lesions like CEOT, odontoma, calcifying odontogenic cyst and dentinogenic ghost cell tumor. After an extensive discussion with the patient’s family, we decided to forego an incisional biopsy and instead proceed to definitive treatment due to the low suspicion for malignancy. This has been the approach at our children’s hospital in the management of pediatric odontogenic neoplasms, and it has its benefits and disadvantages. Benefits of foregoing an incisional biopsy prior to definitive excision includes mitigating the physical and emotional risks of repeated trips to the operating room, while decreasing surgical costs with fewer operations. Children and parents also benefit from fewer missed school days or workdays and decreased total recovery time. Disadvantages of this approach include the lack of a definitive histologic diagnosis at the time of tumor excision and the need to extract teeth involved in the lesion. It is important to stress that the decision of forgoing a diagnostic biopsy should not be taken lightly and is not advised to practitioners without vast experience treating these tumors. Importantly, this should be considered an option only if the tumor has clear signs of benign features. As many of these tumors may be malignant, it is paramount to determine whether the tumor can be treated conservatively or whether *en bloc* resection or hemi-mandibulectomy are advised. We do not recommend this approach when there is a high suspicion for a vascular tumor or a malignant neoplasm. This semi-conservative approach has proven efficacious in our practice over the past 7 years, as evidenced in our series of 15 pediatric patients with odontogenic tumors currently in preparation for publication.

In other very large pediatric tumor centers, the groups also skip biopsies in about 85–90% of mandibular lesions that are ‘clear-cut’ and suspected to be benign based on appearance and relative size (3 cm in a pediatric mandible is much more significant than 3 cm in adult). Hence experience to make such determination is paramount and extreme caution is still warranted.

Since the CEOT recurrence rate in the pediatric population is unknown, we can only extrapolate a recurrence rate based on long term adult data, that indicates a recurrence rate of 12–14% and the potential for malignant transformation [7,16]. Given this recurrence rate, we felt that immediate reconstruction would only cloud appropriate identification of any tumor recurrence. Due to this paucity of data, we decided on a 5 years surveillance period involving cone beam CT (CB-CTs) scan imaging every 6 months for the first two years, followed by annual CB-CTs for the following 3 years.

Even though a 4 year follow up is encouraging in our experience, the literature still reports a well- documented recurrence rate with enucleation of 14–15%, so vigilant observation should be maintained.

## Conclusion

CEOT is a rare odontogenic tumor with a slow growth pattern and a peak incidence in mid-adulthood. Intraosseous CEOT’s have been reported in the pediatric population, but to date never in a child as young as 5 years of age. Because of its rarity, no CEOT treatment consensus exists. We based our semi-conservative management on existing adult CEOT data, as well as on established treatment of other pediatric odontogenic tumors. To avoid multiple surgical procedures, we performed a single stage excision of the lesion with peripheral ostectomy. We defer immediate mandibular reconstruction to allow for adequate surveillance, as hardware and bone graft particles can obscure proper visualization of recurrent tumor foci. As CEOT recurrence in the pediatric population is unknown, close surveillance is paramount in recurrence identification. We recommend 5 years of surveillance, with postoperative imaging biannually for the first two years, then annually for the remainder three years.

## Ethical approval

Due to the retrospective nature of this single case report, Ethics approval or exemption was not required by the Cincinnati Children’s Hospital IRB.
